# Raman Spectroscopy of Multi-Layer Graphene epitaxially Grown on 4H-SiC by Joule Heat Decomposition

**DOI:** 10.1186/s11671-018-2606-2

**Published:** 2018-07-06

**Authors:** Zhiwei Zhang, Weiwei Cai, Rongdun Hong, Dingqu Lin, Xiaping Chen, Jiafa Cai, Zhengyun Wu

**Affiliations:** 1 Department of Physics, Xiamen University, Xiamen, 361005 People’s Republic of China; 2 Jiujiang Research Institute of Xiamen University, Jiujiang, 332000 People’s Republic of China; 3 Fujian Key Laboratory of Semiconductor Materials and Applications, Xiamen, 361005 People’s Republic of China

**Keywords:** Carbon materials, Semiconductors, Raman

## Abstract

We developed a Joule heating decomposition (JHD) method, which applied direct current on the SiC for the epitaxial growth of multi-layer graphene (MLG) films on Si-terminated (0001) face of the high doping 4H-SiC substrate. By this JHD method, the growth time for preparing MLG was only several minutes. Raman spectroscopy was employed to study the influence of the temperature caused by the Joule heating on the quality and the uniformity of the sample. Then, other properties, such as the strain, the layer’s number, and the electric characteristics, of the MLG were studied in details. It was found that the quality of the MLG was substantially dependent on the growth temperature (operation current) and the growth time, while the layer’s number was only dependent on the growth temperature but not the growth time. Finally, less-defect and homogeneous MLG (~ 45 layers) with an area of ~ 12 × 5 mm^2^ could be obtained at a heating temperature of ~ 1470 °C with duration time of 5 min. By using the linear transmission line method, the specific contact resistance of Au and MLG was 5.03 × 10^−5^ Ω cm^2^, and the sheet resistance was 52.36 Ω/sq, respectively.

## Background

Graphene, as a monolayer of carbon (C) atoms with two-dimentional honeycomb lattice, has triggered extensive investigations due to its remarkable mechanical, electronic, and thermal properties in the past decade [1, 2]. Its mechanical and photoelectronic characteristics make it an ideal material for the nanoelectronics, thin-film transistors, transparent electrodes, and printable photoelectronics [3, 4]. Up to today, several techniques for synthesizing large-scale and high-quality graphene have been researched. Mechanical cleavage of graphene from highly oriented pyrolytic graphite produces high-quality but small-sized graphene monolayers [5]. Chemical vapor deposition (CVD) of hydrocarbons is used for epitaxial growth of large-area graphene on the surfaces of transition metals, such as Ni or Cu [6, 7]. Recently, Li and co-workers developed a method to grow graphene flakes directly on silicon with metal free by CVD method, but the size of graphene was still very small [8]. Thermal decomposition of silicon carbide (SiC), by which silicon (Si) atoms are sublimated and a C-rich surface is retained to nucleate an epitaxial graphene (EG) layer, seems to be the promising method for EG production in large area, good quality, and high efficiency [9]. The main advantage of this method is that graphene can be epitaxially grown on the surface of SiC and directly applied to SiC-based optoelectronic and electronic devices without being transferred [10, 11], which could avoid the defects or damages caused during the transfer process of the graphene prepared by the methods of cleavage or CVD.

Recently, several thermal decomposition methods for EG growth have been reported, such as radio-frequency induction heating [12], laser heating [13], and other heating methods [14]. Comparing to these methods, we developed a Joule heating decomposition (JHD) method by applying direct current (DC) on SiC to generate Joule heating on the surface of SiC. By adjusting the DC, heating temperature on the surface of SiC could be modulated from ~ 1230 to 1600 °C or higher. The main advantages of JHD method over other thermal decomposition methods for preparing EG are that the temperature for growing EG on the surface of SiC could be reached in a few seconds and the size of the graphene layer could be produced as large as the size of SiC substrate which was prepared with an appropriate ratio of length and width. Therefore, the JHD method could be regarded as the low-cost and high efficiency method for EG growth on SiC. In this paper, Raman spectra of multi-layer graphene (MLG) epitaxially grown on 4H-SiC by JHD were studied to understand the influences of the operation current, growth temperature, and growth time on the structural and electrical properties of the MLG.

## Methods/Experimental

### Growth of Graphene on 4H-SiC

**Fig. 1:**
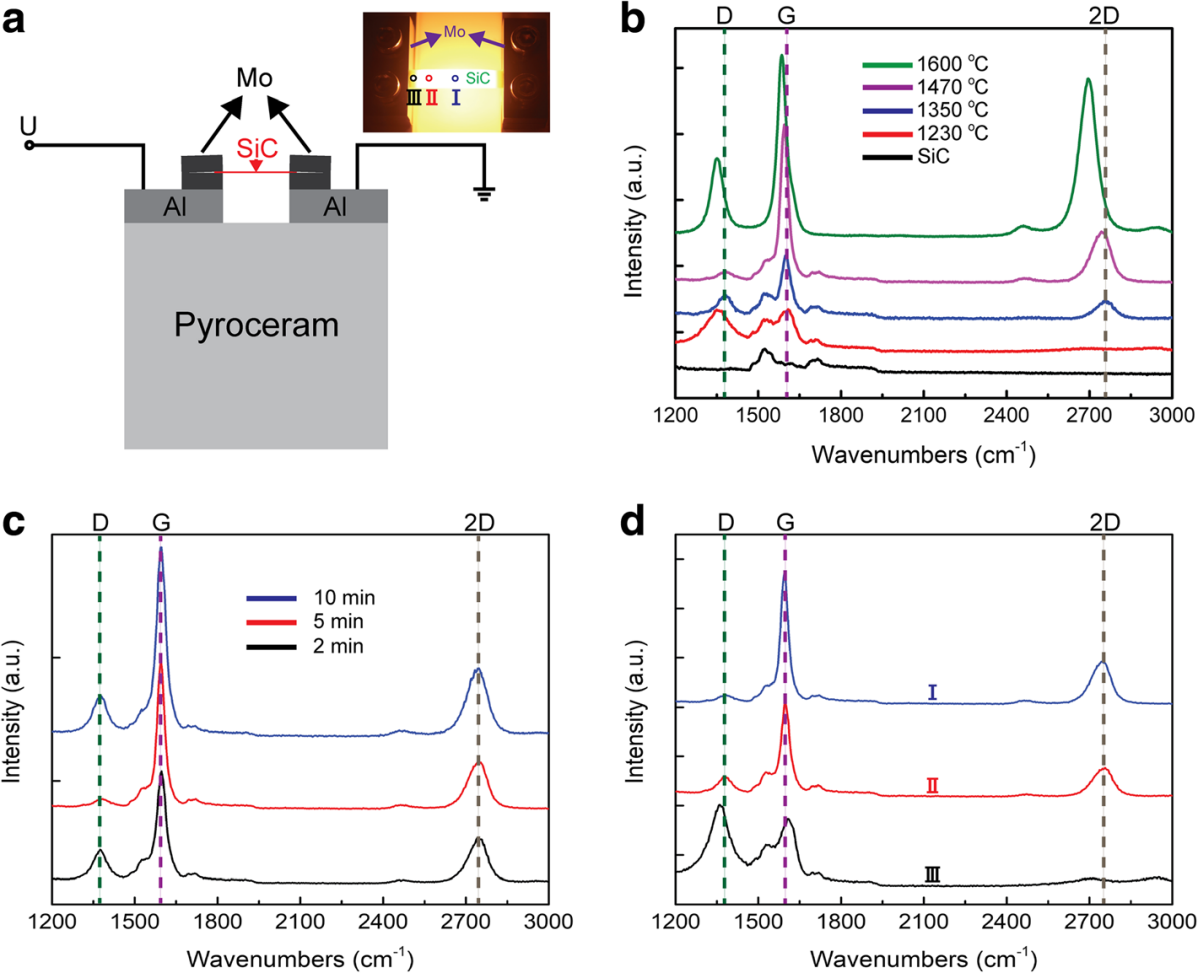
**a** Schematic diagram of the platform for MLG growth by JHD. The inset was the image of the SiC during heating process. **b** Raman spectra of SiC and MLG grown on 4H-SiC (0001) at different growth temperatures for 5 min. **c** Raman spectra of MLG grown on 4H-SiC (0001) at 1470 °C for 2, 5, and 10 min, respectively. **d** Raman spectra characterized from the circled spots A, B, and C marked in the inset of **a** on the same sample. The sample was prepared at 3.24 A for 5 min

Two-inch N-type 4H-SiC (350 μm thick, ~ 0.02 Ω cm) wafers were purchased from SICC Materials Co., Ltd. A custom-made vacuum chamber and a ceramic cube with two aluminum (Al) and four small molybdenum (Mo) electrodes as a heating platform were used for the graphene growth. The wafers were sliced to several pieces of 25 mm × 5 mm substrates by a cutting machine before carefully treated by sonication with methanol, acetone, and ethanol three times, followed by wet-chemical RCA cleaning. After dried with N_2_ flow, put the SiC substrate between Mo electrodes on the heating platform, which was connected to a DC source, as shown in Fig. 1a. Subsequently, the base was placed into the vacuum chamber in which the air pressure will be vacuumed to ~ 10^−6^ Torr, followed by applying a DC on the SiC to generate a large Joule heat. With the DC applied from 2.79 to 3.43 A, the surface temperature of the SiC could be increased highly enough for the growth of graphene. After graphene growth, the samples were cooled down in the vacuum chamber for more than 4 h before characterization.

### Sample Characterization

SiC substrates were cut by an automatic grinding wheel cutting machine, ZSH-406. The temperatures of sample’s surface were measured by the MI16MB18 infrared thermometer from Sensortherm. Raman spectroscopy was carried out by WITec alpha 300RA confocal microscope system comprising of a laser with 488 nm wavelength and a UHTS 300 spectrograph (600 lines/mm grating, 30 cm focal length) coupled with a Peltier-cooled CCD detector. An atomic force microscope (AFM) (SPA-400) was used to characterize the morphology of the MLG before and after etching. The etching of MLG was performed by the inductively coupled plasma (ICP) 98 A with 30 sccm of O_2_ for 60 s. The Au was deposited onto the MLG by evaporation using the same system as the growth process. A Au wire was heated to evaporate slowly by applying a DC on it, which was fixed on top of the MLG sample. With lithography, we prepared Au-graphene contact and measured the IV properties by the linear transmission line method (LTLM). The IV was carried out by using a Keithley 2410 SourceMeter and a Keithley 6514 system electrometer at room temperature.

## Results and Discussion

Four MLG samples were prepared by applying different DCs of 2.79, 3.05, 3.24, and 3.43 A on the SiC substrates, and the DCs were kept stable for 5 min during the synthesis of graphene. With the increase of the DCs, the temperatures at the center of substrates were ~ 1230, 1350, 1470, and 1600 °C, respectively. After the growth of MLG was completed, the samples were investigated by Raman spectroscopy. As shown in Fig. 1b, several peaks corresponding to graphene were observed, which were identified by three main bands: (i) the defect-induced D band at frequency of ~ 1370 cm^−1^, (ii) the in-plane vibrational G band at frequency of ~ 1600 cm^−1^, and (iii) the two-phonon 2D band at frequency of ~ 2750 cm^−1^ [15]. Compared to a single-layer micromechanical cleavage graphene (MCG), an important observation was that the G (~ 1600 cm^−1^) and 2D (~ 2750 cm^−1^) bands of MLG shift significantly toward higher frequency from those of G (1580 cm^−1^) and 2D (2673 cm^−1^) of MCG [16]. There might be several reasons that caused the significant shifts of G band (~ 20 cm^−1^) and 2D band (~ 77 cm^−1^). Ni illustrated how the strain effect of the epitaxial graphene on 6H-SiC changed the lattice constant of graphene, and further affected the Raman frequencies [16]. Others have reported that the doping might cause the blue-shift of G and 2D peaks [17–19], but the effect was very weak comparing to the one aforementioned. Here, the blue-shift of G and 2D bands could be attributed to the strain effect which was caused by the lattice mismatch of graphene and SiC substrate [16]. From Fig. 1b, we observed the appearance of corresponding G band and D band of graphene from the red spectrum, which was taken from the MLG sample prepared at ~ 1230 °C. The high value of *I*_D_ (intensity of D band) divided by *I*_G_ (intensity of G band) (*I*_D_/*I*_G_) and no obvious evidence for 2D band indicated lots of defects and a poor crystallinity of graphene. The reason might be that C atoms could not obtain enough kinetic energy to process the reconstruction of the graphene well at such low growth temperature [20]. By increasing the heating temperature to ~ 1350 °C, the value of *I*_D_/*I*_G_ decreased from ~ 1.01 to ~ 0.38, which indicated the MLG had a lower ratio of defects. The symmetric 2D band with a full width at half maximum (FWHM) ~ 72 cm^−1^ further demonstrated the crystallization of MLG and its better quality. And, the low Raman intensity of SiC has proved that the samples we have prepared were the multi-layer graphene [21]. With the growth temperature further increased to 1470 °C, the *I*_D_/*I*_G_ continued to decrease to ~ 0.06, indicating the number of defects was further reduced. Moreover, the 2D band had a slight red shift. We assume there might be a strain relief in the interface between MLG and SiC as more graphene layers formed at higher Joule heating temperature [16]. We also investigated the MLG that was prepared at ~ 1600 °C with Raman spectroscopy. However, a higher *I*_D_/*I*_G_ (~ 0.43) was observed, indicating an increase of defects. Our hypothesis was that it might originate from the high graphitization rates in the out of equilibrium vacuum sublimation process, and thus, it caused more surface dislocations or corrugations on the surface of MLG [14]. Besides, further red shifts of D, G, and 2D bands were observed, which meant more strain relief, and thus, more graphene layers were synthesized [16].

We then focused on the influence of JHD process time on the growth of MLG. As the *I*_D_/*I*_G_ of the MLG grown at 1470 °C was lowest, three samples were prepared at the DC of 3.24 A (~ 1470 °C) for 2, 5, and 10 min, respectively, and Raman spectra were shown in Fig. 1c. The *I*_D_/*I*_G_ of the MLG grown for 5 min was about 0.06, which was lower than the other grown for 2 min (~ 0.41) and 10 min (~ 0.29), indicating that MLG grown for 5 min had the fewest defects. The reason might be that 2 min was too short for C atoms to reconstruct homogeneous graphene layers, and graphene defects such as discontinuity, inhomogeneity, and stacking disorder occasionally appeared. However, 10 min might be too long for MLG growth, as they would be affected by the residual gases in the chamber and thus generate defects [22]. As time increased, no red shift of G or 2D peak position was observed from Fig. 1c, indicating the strain between graphene layers and the substrate should be almost the same for these samples. The unchanged strain might be that the number of graphene layers was barely increased, as the *I*_G_/*I*_2D_ were almost the same (2.7 for 2 min, 3.0 for 5 min, and 2.8 for 10 min) and the *I*_SiC_/*I*_G_ was barely change, where *I*_SiC_ is the intensity of Raman band (at ~ 1520 cm^−1^) for 4H-SiC [21].

Due to the thermal conductivity difference, the Joule heating power at the contact surface of SiC and Mo electrodes would escape faster. In that case, the substrate’s center would obtain the highest temperature during the JHD process, while if the spot was closer to the Mo electrodes, the heating temperature would be lower. Therefore, Raman spectroscopy was used to characterize the MLG from different spots (as shown in the inset of Fig. 1a) on the sample prepared at the DC of 3.24 A, and the results were shown in Fig. 1d. The distances are about 3 mm between position C and B, and about 6 mm between positions B and A. Raman spectra of A and B showed rather a low value of *I*_D_/*I*_G_, along with symmetric 2D bands, which indicated few defects. The barely change of *I*_G_/*I*_2D_ and *I*_SiC_/*I*_G_ also proved similar layer’s number of MLG between these two positions. Furthermore, no clear Raman shifts of the G and 2D band also demonstrated the homogeneity of MLG. Therefore, we could synthesize an area of ~ 12 × 5 mm^2^ MLG with good uniformity of graphene layers by the JHD method.

**Fig. 2:**
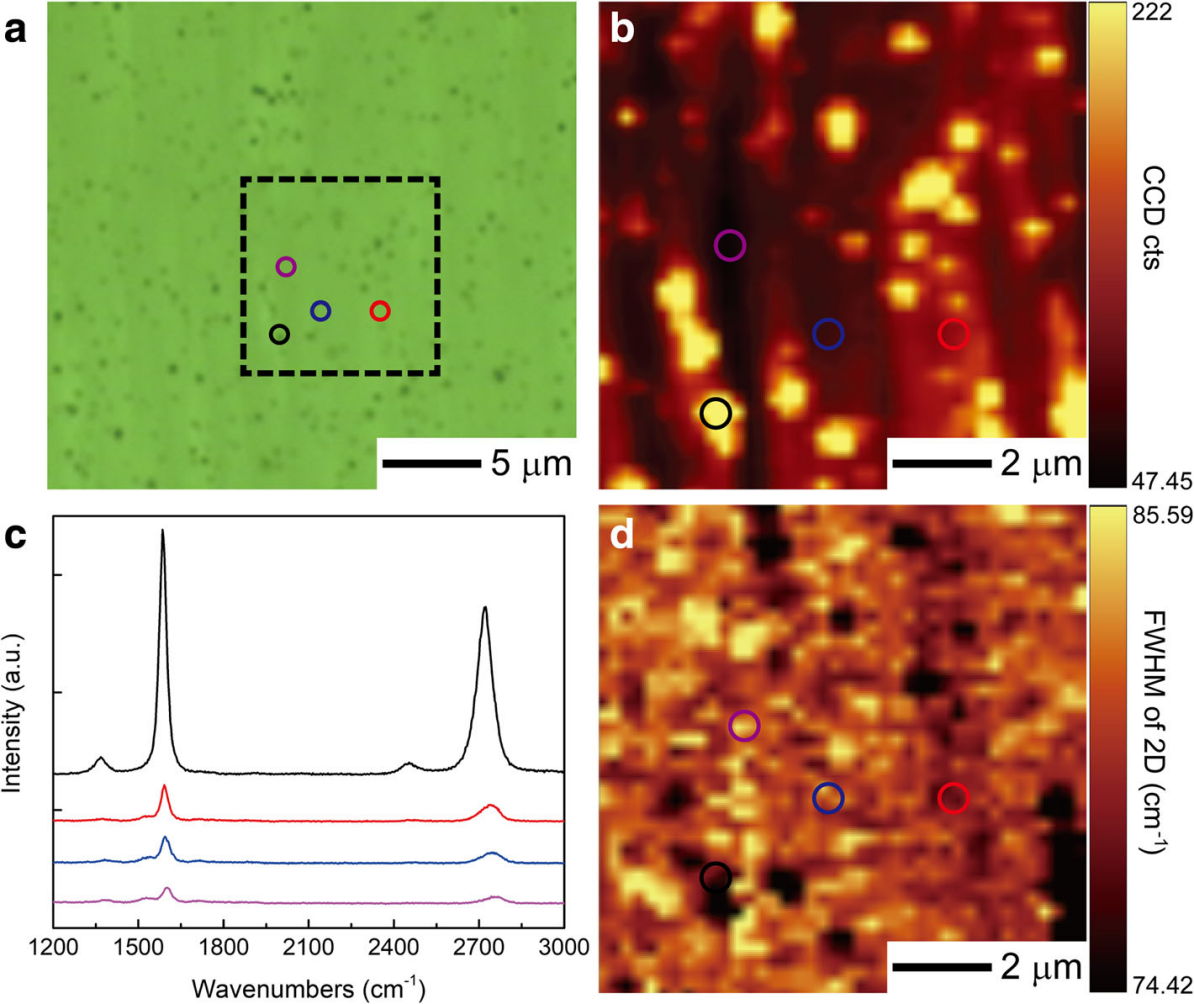
**a** Optical image of MLG sample which was prepared at 3.24 A for 5 min and characterized from the center. **b** Raman mapping for the intensity of 2D band from the marked area in dashed square in **a**. **c** The Raman spectra from the marked circles in **b**. **d** Raman mapping for the FWHM of 2D band

To further study the uniformity of the MLG, Fig. 2a illustrated the optical image of the sample characterized from the area A in the inset of Fig. 1a. It is shown in Fig. 2a that most of the color contrast of the surface was quite even except for some dark dots. We found these dark dots had the highest intensity of 2D band, as shown in the Raman mapping of Fig. 2b. Figure 2c demonstrated the Raman spectra of the corresponding area marked in circles in Fig. 2b with a different color. It also showed that the intensity of G and 2D bands from the dark dots (black circle) were much higher than the other area. Besides, the peak position of both G and 2D bands was slightly red shifted. The hypothesis was that the formation of graphene would prefer sites of screw dislocations or other defects (the dark dots in our work) on the surface of SiC [23], and the speed of decomposition of SiC, as well as the growth of graphene, would be faster than the other area. Figure 2d demonstrated the full width at half maximum (FWHM) of 2D band, which was rather uniform except for regions where defects of SiC were present.

**Fig. 3:**
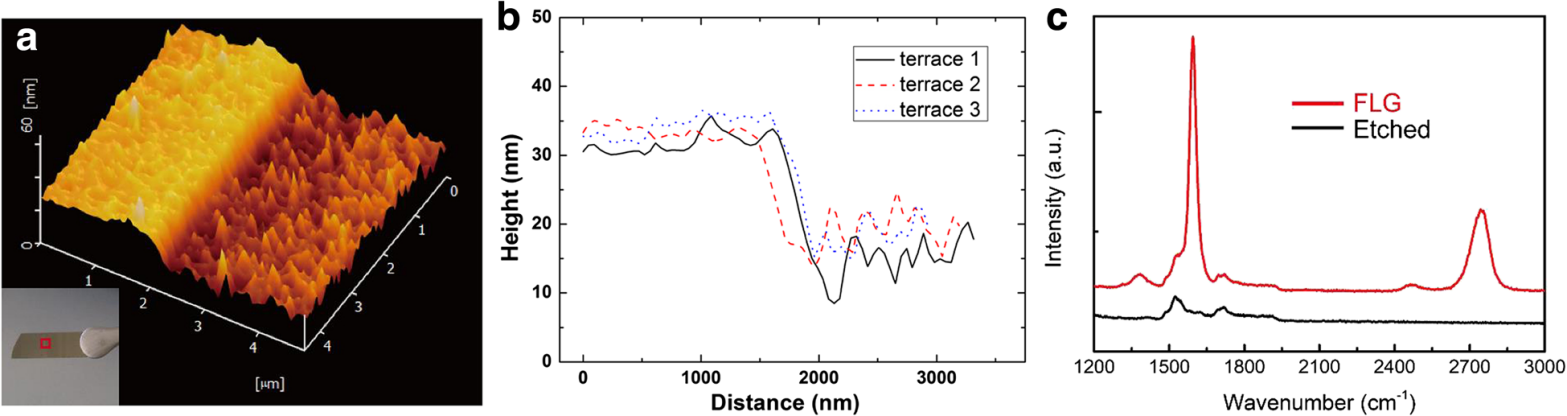
**a** AFM image of MLG with half etched by ICP-etching which was taken in the red square of the inset. The inset was the image of the MLG sample, and the light part was covered by MLG. The MLG was synthesized at 1470 °C for 5 min. **b** height profiles of the terrace at different position on the AFM image. The average height of the terrace is ~ 15.46 nm. **c** Raman spectra of the sample in **a**, the red and black spectra were corresponding to the sample before and after etching

To investigate the number of layers of the graphene that we prepared at ~ 1470 °C for 5 min, we use AFM to characterize the MLG sample after the ICP-etching, as shown in Fig. 3a. Etched with O_2_, there was a terrace between MLG and the etched part. The inset in Fig. 3a also demonstrated the difference of contrast, while the light part was unetched and the dark part was etched. And the height profiles of the terrace at different positions on the AFM image were illustrated on Fig. 3b. To further confirm the existence of graphene after etching, Raman spectra were taken at the spots with and without ICP-etching, as shown in Fig. 3c. The unobvious D, G, or 2D bands proved that the graphene had been etched away completely. We then measured the average height difference between the MLG and the etched part thought the height profiles, and the value was ~ 15.46 nm, which meant the number of graphene layers was ~ 45 (the interlayer spacing was ~ 0.34 nm) [24]. Besides, the root-mean-square (RMS) value increased from 0.84 to 2.79 nm after ICP-etching, which might be due to the difference of decomposition speed of SiC caused by the defects and thus generate a rough surface of SiC after growth of graphene.

**Fig. 4:**
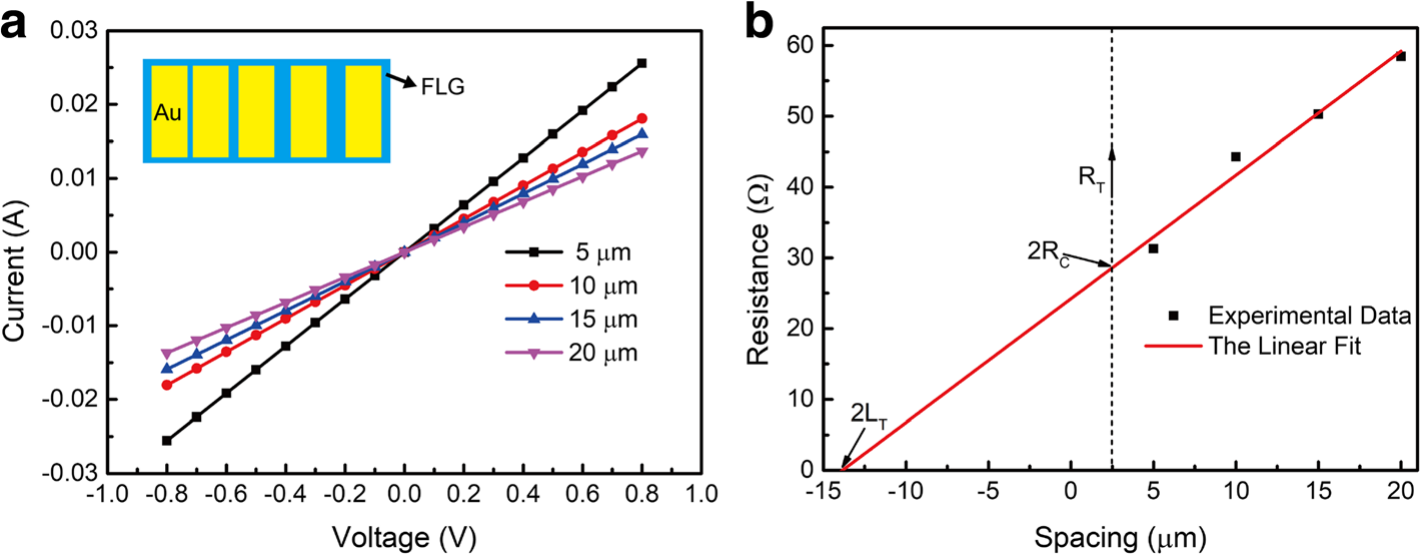
**a** The IV properties of the Au-graphene-Au contact. The inset is the schematic diagram of LTLM. **b** The linear fit of the total contact resistance of Au ohmic contact as a function of contact pads distance from 5 to 20 μm

We then investigated the electric properties of the MLG (synthesized at ~ 1470 °C for 5 min). At room temperature, we measured the IV properties of the adjacent Au electrodes of the LTLM, as shown in Fig. 4a. According to the equations [25],

1 $$ {R}_{\mathrm{T}}=\left({\rho}_{\mathrm{s}}/Z\right)d+{2R}_{\mathrm{C}}\approx \left({\rho}_{\mathrm{s}}/Z\right)\left(d+{2L}_{\mathrm{T}}\right) $$

2 $$ {\rho}_{\mathrm{c}}={\rho}_{\mathrm{s}}{L}_{\mathrm{T}}^2 $$

While *R*_T_ is the total resistance, *ρ*_s_ is the sheet resistance, *R*_C_ is the contact resistance, *ρ*_c_ is the specific contact resistance, *Z* is the width of the MLG (40 μm), *d* is the space between the Au electrodes (5, 10, 15, and 20 μm, respectively), and *L*_T_ is the length of transmission line for electricity. By the linear fit of the experimental data, as shown in Fig. 4b, we could get *R*_*C*_ and *L*_*T*_. According to the Eqs. (1) and (2), *ρ*_s_ and *ρ*_c_ were calculated to be 52.36 Ω/sq and 5.03 × 10^−5^ Ω cm^2^, respectively.

## Conclusions

In summary, a convenient JHD method by applying DC power on SiC in vacuum (~ 10^−6^ Torr) was developed to grow multi-layer epitaxial graphene directly on 4H-SiC (0001) substrate. By optimizing the growth conditions, large-area (12 mm × 5 mm) and low-defect MLG with a good homogeneity could be obtained by heating SiC at ~ 1470 °C for 5 min, as Raman spectroscopy showed the lowest *I*_D_/*I*_G_. AFM result illustrated that the MLG was ~ 45 layers thick. The MLG also demonstrated a good ohmic contact with Au electrode. In our further works, epitaxial SiC on the SiC substrate will be selected for the MLG growth by JHD. Also, the low defect of SiC epitaxial layer would be another advantage for preparing MLG with high homogeneity and quality. Besides, the confinement control method such as introducing an inert gas will be employed into the JHD growth to adjust the growth rate, improve the quality, and obtain a higher homogeneity. The graphene produced by JHD method could be promising in the applications of SiC-based photoelectronic devices in the future.

## Abbreviations

AFM: Atomic force microscope
Al: Aluminum
C: Carbon
CVD: Chemical vapor deposition
DC: Direct current
EG: Epitaxial graphene
FWHM: Full width at half maximum
ICP: Inductively coupled plasma
*I*_X_: Intensity of X band
JHD: Joule heat decomposition
LTLM: Linear transmission line method
MCG: Micromechanical cleavage graphene
MLG: Multi-layer graphene
Mo: Molybdenum
SiC: Silicon carbide
